# Spatiotemporal dynamics of random stimuli account for trial-to-trial variability in perceptual decision making

**DOI:** 10.1038/srep18832

**Published:** 2016-01-11

**Authors:** Hame Park, Jan-Matthis Lueckmann, Katharina von Kriegstein, Sebastian Bitzer, Stefan J. Kiebel

**Affiliations:** 1 Department of Psychology, Technische Universität Dresden, Dresden, Germany; 2 Department of Neurology, Max Planck Institute for Human Cognitive and Brain Sciences, Leipzig, Germany; 3 Neural Computation and Behaviour Group, Max Planck Institute for Biological Cybernetics, Tübingen, Germany; 4 Department of Psychology, Humboldt Universität zu Berlin, Berlin, Germany; 5 Neural Mechanisms of Human Communication Group, Max Planck Institute for Human Cognitive and Brain Sciences, Leipzig, Germany

## Abstract

Decisions in everyday life are prone to error. Standard models typically assume that errors during perceptual decisions are due to noise. However, it is unclear how noise in the sensory input affects the decision. Here we show that there are experimental tasks for which one can analyse the exact spatio-temporal details of a dynamic sensory noise and better understand variability in human perceptual decisions. Using a new experimental visual tracking task and a novel Bayesian decision making model, we found that the spatio-temporal noise fluctuations in the input of single trials explain a significant part of the observed responses. Our results show that modelling the precise internal representations of human participants helps predict when perceptual decisions go wrong. Furthermore, by modelling precisely the stimuli at the single-trial level, we were able to identify the underlying mechanism of perceptual decision making in more detail than standard models.

Perceptual decision making is a core aspect in everyday cognition. In difficult perceptual situations, for instance when driving a car through heavy rain and trying to read a traffic sign, perceptual decision making can be error prone. Standard models assume that these errors are either due to a high noise level in the sensory input (raindrops on the windshield), or to internal brain processes (neuronal noise) or to a mixture of both^1^,^2^,^3^.

In the laboratory, perceptual decision making is usually investigated by linking the perception of incoming sensory information to making a choice among several alternatives^4^,^5^,^6^,^7^,^8^, often under time pressure. For example, in the widely-used random dot motion task (RDM), participants are required to report the net direction of a cloud of moving dots within a certain time frame^9^. The difficulty of such a decision depends on the amount of noise in the stimuli, i.e. the percentage of coherently moving dots in the cloud of dots^10^,^11^. The two current standard approaches to modelling behavioural data of such perceptual decision making experiments are the drift diffusion model (DDM)^12^,^13^ and the nonlinear attractor model^14^,^15^. These models analyse average measures of the behavioural data (e.g. performance expressed as percentage of correct decisions and averages of the reaction times across trials). The implicit assumption of these analyses is that behaviour can be understood by the average effect of sensory noise on decision making performance^12^.

What standard models cannot explain however, is how each single decision depends precisely on the specific noise pattern in that particular input stimulus. By only looking at the average performance over many trials, one cannot tell what caused a particular incorrect decision: In the example above, the raindrops might be in one instance covering more informative parts of the traffic sign than in others. Following this intuitive notion, we hypothesized that errors in perceptual decisions can be explained better based on the precise pattern of sensory input noise, as compared to using the average noise level. Such a finding would mean that one can analyse perceptual decision making data in high detail, and thereby enable more precise inference about the underlying mechanisms of decision making.

To test this hypothesis, we developed a novel paradigm which enables tracking of the full details of what the observer has seen. This is necessary in order to test whether the participant utilizes the exact spatio-temporal dynamics of the input. We tested this by comparing a model which is informed about the actual trial-wise spatio-temporal noise details of the input stimuli; the exact input model (ExaM), and another model which does not include this information but relies, as standard models do, on average information about the stimuli plus noise as input. This model is mathematically equivalent to the pure drift-diffusion model (DDM)^16^, which is a standard model for perceptual decision making. We will therefore call this the DDM-equivalent model. Similarly constrained models as ExaM have been long developed in mathematical psychology^17^, and several studies in decision making used this technique to model traditional psychology paradigms, e.g.^18^,^19^,^20^. Here we applied this approach to computational models of perceptual decision making and demonstrate the implications based on systematic analyses.

We expected two key findings. First, the ExaM will be able to explain more precisely and on a single-trial basis, how human participants make their perceptual decisions, as compared to the DDM-equivalent model. Second, this increase in modelling precision can be used to answer more detailed questions about the underlying mechanism of perceptual decision making. To show this, we addressed the question whether participants use a specific mechanism to respond within a specific time window. One standing hypothesis is that participants dynamically lower their internal criterion of how much information is needed to commit to a decision by reducing their decision boundaries^21^,^22^. Neurophysiological experiments have found evidence that monkeys do employ this so-called collapsing bound model (or, similarly, the urgency signal model)^21^,^22^,^23^. However, in humans, there is less clear evidence presumably because the effect of time-pressure on behavioural data is small^24^,^25^. We hypothesized that a more precise model, as proposed here, should be able to detect whether human participants are indeed using a collapsing bound when making perceptual decisions.

## Results

### Task and Behavioural Data

**Figure 1:**
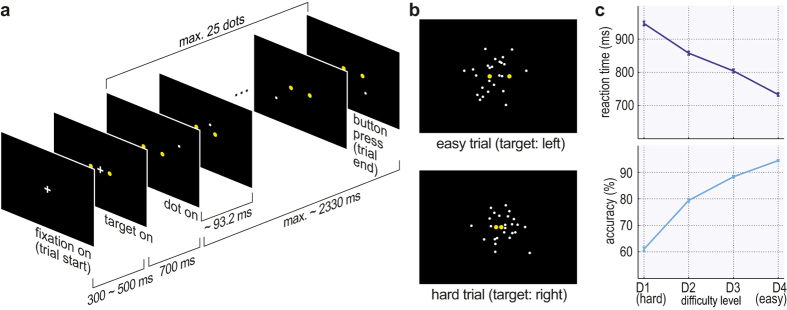
Experimental design and behavioural data on group level. (**a**) The fixation cross is shown for a duration jittered between 300 and 500 ms, followed by the onset of two target dots (yellow) for 700 ms. After that, a single white dot starts to show, so that the participant observes a single white dot relative to two yellow targets. The position of the white dot changes every 93 ms. A trial ends when the participant makes a decision or when a maximum of 25 dots (~2330 ms) has been shown. The task is to decide fast and accurately around which of the targets the dot is moving. (**b**) Illustration of stimulus shown to participants summarised over time: The positions of the two yellow dots (targets) is fixed for one trial. The white dots are superimposed for all frames, to depict the variance of the dot trajectory. In the experiment, participants see each dot as a single frame, consecutively. The distance between the targets determines the difficulty of a trial, as the dot positions are drawn from a Gaussian with the correct target position as mean. (**c**) Reaction time and accuracy (as percentage of correct trials) averaged over all participants for four difficulty levels (D1–D4). Error bars are standard error (SE) over all participants, all non-timed-out trials.

We used a novel behavioural paradigm to investigate perceptual decision making. Human participants (n = 24) saw a single white moving dot, randomly jumping around two yellow target dots on a black computer screen. The white dot locations were sampled from a Gaussian distribution with its mean on one of the two targets with fixed standard deviation. The white dots jumped to a different location on the screen every 93.2 ms, for a maximum of 25 jumps, i.e., 2.33 s. Participants were told that the white dot represents a bee. The task was to decide whether the bee is coming from the left beehive or the right (the two yellow target dots) (Fig. 1a). The task had four difficulty levels, depending on the distance between targets (Fig. 1b). We recorded the decision (left/right, or timed-out) and reaction time (RT) for each trial. The participants were told to be as accurate and as fast as possible, and were given a monetary reward for overall high accuracy and a small number of timed-out trials. As expected, for higher difficulty levels, the accuracy decreased and RT increased (Fig. 1c). The mean accuracy over participants was between 94.4% (standard error (SE) = 0.33) at the easiest difficulty level (D4) and 61.0% (SE = 0.71) at the hardest difficulty level (D1). The corresponding mean RTs were 733 ms (SE = 4.6) and 947 ms (SE = 6.0) (Fig. 1c). The amount of timed-out trials was small (0.2% in total).

### Models

#### DDM-equivalent model vs. Exact Input Model

**Figure 2:**
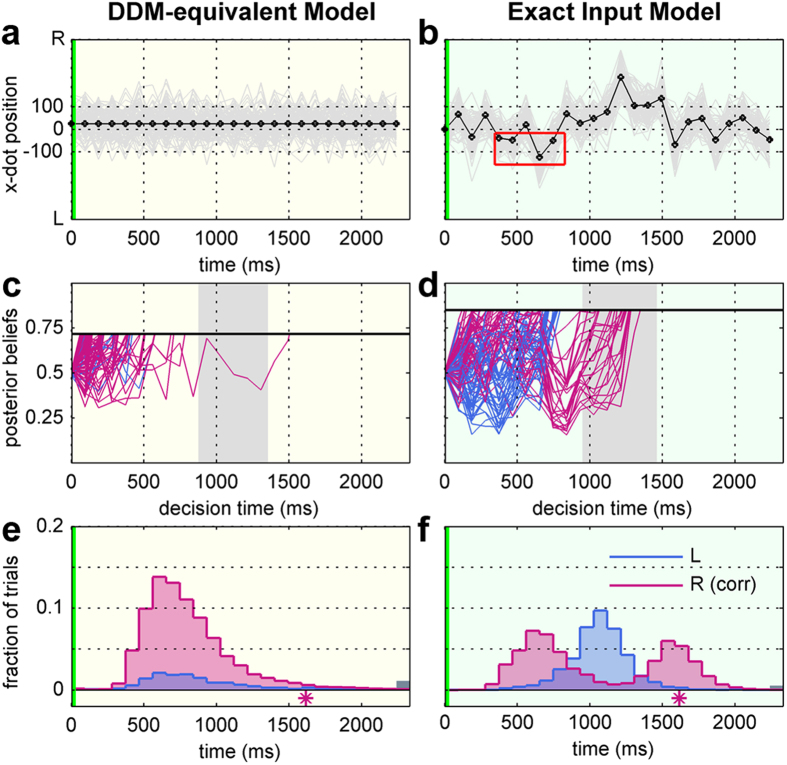
Single trial evidence accumulation and predicted RT distribution for DDM-equivalent model and exact input model (ExaM) from the second hardest difficulty level (D2) from participant 20. (**a**) The input to the DDM-equivalent model: the mean of the correct target (black line indicating right target in this trial) with Gaussian noise (grey lines). (**b**) The input to the ExaM: exact dot position added with Gaussian noise. Red box indicates dots leading to an early left decision. (**c**) Posterior beliefs as in accumulated evidence, using the parameter estimates for participant 20, D2 under the DDM-equivalent model over 100 iterations. The model mostly predicts “right” (red) decisions at early RTs. In the experiment, the participant decided for the “right” alternative. The grey background shading is an interval defined by participant RT minus 95% quantile interval of the NDT. Black horizontal line is the bound. Here, the process is in the decision time frame (without NDT). (**d**) Same as (**c**) for ExaM. (**e**) Corresponding predicted RT distribution for DDM-equivalent model. Asterisk is participant response. The grey bar at the end indicates timed-out trials. (**f**) Same as (**e**) under ExaM. Green vertical lines in (**a,b,e,f**) indicates stimulus onset.

We used a recently developed Bayesian model^16^ that consists of: (1) stimulus input to the model, (2) generative model(s) of the stimuli, (3) Bayesian inference as evidence accumulation and (4) a decision criterion. Depending on how we modelled the input, we obtained two different model instances, i.e., the DDM-equivalent model and the ExaM. Both models are exactly the same except for the input to each model: In the DDM-equivalent model, we obtained the equivalent to the pure DDM by modelling the input as the constant, correct target position (yellow dot) with random noise^16^ (Fig. 2a). In the ExaM, we used as input the exact white dot positions (the bee locations) plus some noise caused by sensory processing (Fig. 2b). This models the time-varying sensory input seen by the participant during each trial. The Bayesian modelling technique used here computes after each update of the white dot position what the participant should compute as evidence for a decision. This is the so-called posterior beliefs over the two decision alternatives (left/right) Fig. 2c,d. The decision is made when the posterior belief for one of the alternatives exceeds a bound (for details on the models, see Material & Methods).

To estimate the parameters of the two models for each participant, we used an advanced inference scheme called Expectation Propagation – Approximate Bayesian Computation (EP-ABC)^26^. We used seven free parameters that are typically used in modelling perceptual decision making^12^,^16^. (1) Noise standard deviation (SD): the amount of noise in the perceptual process in the brain, (2) the mean and (3) standard deviation of the Gaussian which is transformed to the log-normal distribution for the non-decision time (NDT), i.e., the portion of the RT which is irrelevant to the decision process (e.g., perceptual encoding, motor preparation) (see Material & Methods), (4) The bound: the amount of evidence required to commit to a decision, (5) Prior: the bias of a participant for a specific alternative, (6) Lapse probability: the fraction of trials with a random response, and (7) timed-out (TO) lapse probability: the fraction of timed-out trials within the lapse trials (for a complete list of parameters, see Material & Methods). We estimated the parameters from the behavioural data (reaction time and decision for each single trial) for each participant and each difficulty level (for details see Material & Methods).

We found two main differences between parameters fitted by the DDM-equivalent model and the ExaM (Table 1): As compared to the DDM-equivalent model, the ExaM has for all difficulty levels (i) significantly lower noise parameter estimates (~48 pixels lower over conditions) and (ii) significantly shorter non-decision times (~40 ms shorter over conditions) (NDT mode, see Material & Methods). These differences in parameters indicate that with the ExaM the variability in the participants’ behavioural data was explained by using less neuronal noise and a shorter unspecific decision delay, as compared to the DDM-equivalent model. This means that the ExaM is able to explain more variability of the data by the actual decision making mechanism, as opposed to unspecific noise and processing delays.

#### Input Stimuli and Evidence Accumulation

How is this noise reduction achieved by the ExaM? In Fig. 2a,b the sensory input for the DDM-equivalent model and ExaM is shown for an illustrative single trial. For the ExaM, the dot jumps around the right target but then (due to the random movement of the dot) jumps to the left side for several time points between 400 ms and 900 ms (red box in Fig. 2b).

Figure 2c,d show the evolution of the posterior beliefs for both models. The DDM-equivalent model mostly predicts a decision for the right, correct target (Fig. 2c). In contrast, the ExaM gives more specific predictions reflecting the dynamics in the actual sensory input over time (Fig. 2d). In this example trial, the participant’s (correct) response lies within the RT distribution predicted by the ExaM (Fig. 2e,f, asterisks below histograms). Interestingly, the ExaM also predicted that the participant may have responded earlier with the correct response, followed by a time period where the incorrect response would have been likely. This dynamic aspect of the ExaM predictions makes the model diverge from the predictions of the DDM-equivalent model, which (always) predicts a uni-modal RT distribution (Fig. 2e). This enables the ExaM to explain more variability in actual decision behaviour. While this is an exemplary single-trial result, this difference between models is the basis for the following results.

### Model Comparison

**Figure 3:**
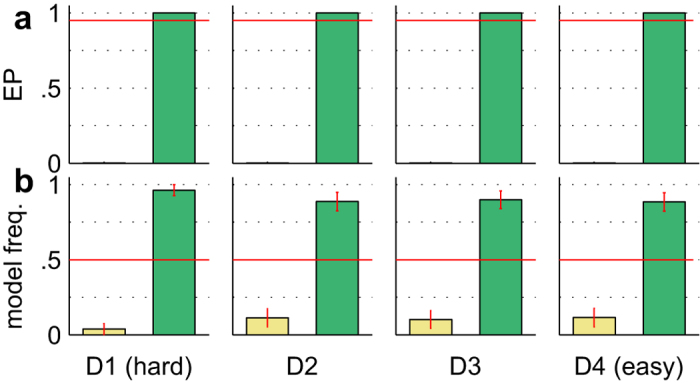
Bayesian Model Selection results for different input models with standard accumulation. (**a**) Protected exceedance probability (EP) (belief of one model being more likely than any other model) between the DDM-equivalent model (yellow bars) and exact input model (green bars). The cut-off probability for the exceedance probability is typically set at 0.95 (red line). (**b**) Posterior model probability (expected probability of obtaining the model when randomly selecting a participant). The red line is the null frequency profile over models, i.e., chance frequency level^28^. Error bars are standard deviation over participants.

We used Bayesian Model Selection^27^,^28^,^29^ and the posterior predictive log-likelihood (PPL) to formally compare the ExaM and DDM-equivalent model. Bayesian Model Selection is based on the so-called protected exceedance probability and the posterior model probability^28^,^29^. We found strong evidence for the ExaM compared to the DDM-equivalent model (Fig. 3a): the protected exceedance probability was approximately 1.00 for all difficulty levels, which means that the belief that the ExaM is more likely than the DDM-equivalent model is nearly 100%. The posterior model probability of the ExaM was on average above 90% (Fig. 3b). This means that the probability that the ExaM generated any randomly selected participant data is above 90%. These two results strongly in favour of the ExaM cannot be explained by different amounts of parameters in the model, because both models had exactly the same number of parameters; the only difference was how the input stimuli were modelled. The results indicate that using the exact input seen by participants results in a better model than relying on the average input assumptions made by the standard model, the DDM.

**Figure 4:**
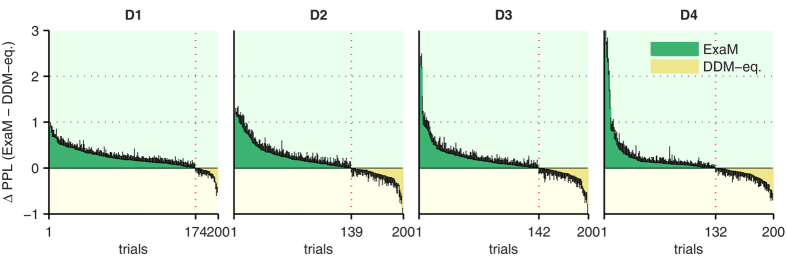
Comparing differential posterior predictive log-likelihoods (ΔPPL) between DDM-equivalent model and exact input model (ExaM), with standard accumulation. The difference in posterior predictive log-likelihoods (PPL) for each condition and each trial, averaged over 24 participants. PPLs of the DDM-equivalent model were subtracted from PPLs of the ExaM. A positive value (green) indicates that the ExaM has higher PPL than the DDM-equivalent, and a negative value (yellow), the opposite. The vertical black bars are the standard error of mean. The vertical red dotted line indicates the number of trials which the ExaM was better, with the number of trials indicated below.

Next, to compare the two models at the single trial level, we calculated the PPL for each trial and each participant. The PPL quantifies how likely a participant’s response is under a given model after fitting the parameters. As we have shown the same 800 trials to each participant we can average the trial-specific PPL, over participants. Figure 4 shows that a larger number of trials, for each difficulty level, has a higher PPL for the ExaM than for the DDM-equivalent model. This is most obvious for the most difficult condition (D1), for which the stimuli display the highest levels of random movements. This further substantiates that behavioural responses can be explained better if one informs the model about the exact noise pattern of the stimulus.

**Figure 5:**
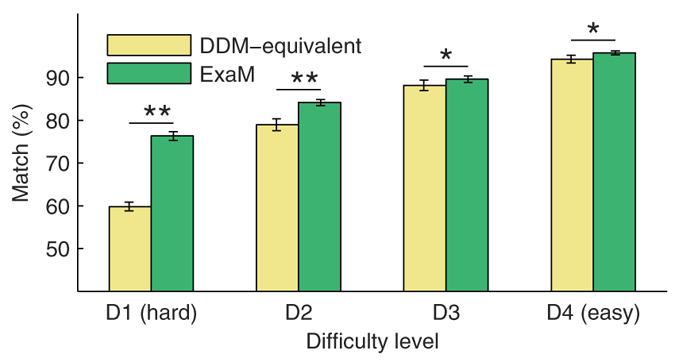
Model comparison using point predictions. For each participant the posterior parameter distributions were used to generate predictions of decisions (decision for left or right target). The fraction of matched decisions over 800 trials, averaged over the 24 participants, for both DDM-equivalent model and exact input model (ExaM) are plotted. Error bars indicate standard error over 24 participants. Matches were significantly higher for the exact input model for all conditions (*p < 0.05, **p < 0.01, paired t-test over 24 subjects). Differences between the two models from D1 to D4 were 16.5%, 5.2%, 1.5%, and 1.4%, respectively. Yellow: DDM-equivalent model, green: exact input model.

To further show the difference between the two models, we calculated point-predictions for matches between model and participant responses (Fig. 5). We calculated the most probable decision for each trial (see Material & Methods) based on the PPLs, and compared the model choice to actual participant choice for both DDM-equivalent model and ExaM. Figure 5 shows that for each of the four difficulty levels, the ExaM predicted the participants’ decisions better than the DDM-equivalent model. Similarly, the ExaM provides for more accurate RT point estimates (Fig. S1). Again, as with the PPL results, the difference between the two models was more prominent in the most difficult conditions. Critically, we also found that the ExaM explained significantly more errors made by participants, as compared to the DDM-equivalent model (Table 2).

### Decision Making Mechanisms

**Figure 6:**
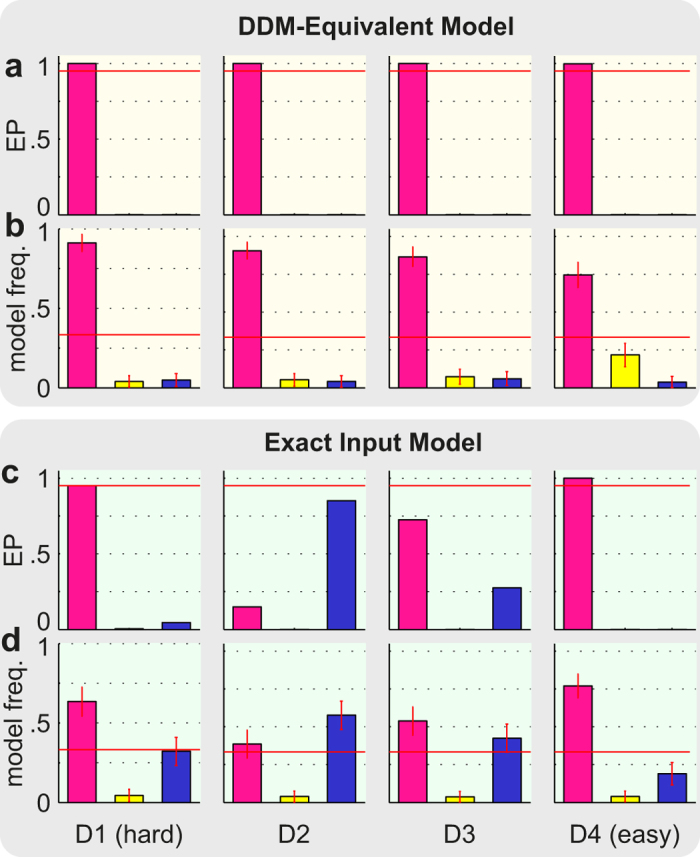
Bayesian model selection results for different accumulation mechanisms for the DDM-equivalent and exact input model. (**a**) Protected exceedance probability (EP) (belief of one model being more likely than any other model) between the three accumulation mechanisms: (i) standard accumulation (pink bars) as tested for in Fig. 3, (ii) leaky integration (yellow bars), and (iii) collapsing bound mechanism (dark blue bars). The cut-off probability for the exceedance probability is typically set at 0.95 (red line). (**b**) Posterior model probability (expected probability of obtaining the model when randomly selecting a participant). The red line is the null frequency profile over models, i.e., chance frequency level^28^. (**c**) Same format as in (**a**) but for the ExaM. (**d**) Same format as in (**b**) but for the ExaM. Error bars are standard deviation over participants. The model comparisons between the accumulation mechanisms were performed within the DDM-equivalent (**a,b**), and exact input (**c,d**) model, respectively.

**Figure 7:**
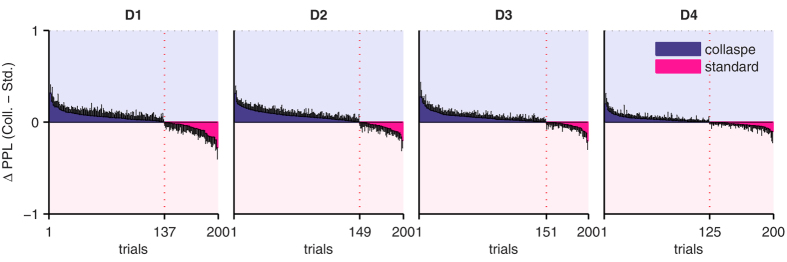
Comparing differential posterior predictive log-likelihoods (ΔPPL) between standard accumulation and collapsing bound models per participant, in the exact input model (ExaM). Format as in Fig. 4, but for collapsing bound model and standard accumulation model, both within the ExaM.

Next we tested our hypothesis that the ExaM outperforms the DDM-equivalent model in providing evidence for a collapsing bound decision-making mechanism. To do this, we modelled and compared, for both models, three different mechanisms that have been suggested for perceptual decision making: the standard evidence accumulation employed above, the so-called leaky accumulation^30^,^31^, and the collapsing bound model^21^,^22^,^32^. For the DDM-equivalent model, the Bayesian Model Selection result showed no evidence for the collapsing bound model; the standard evidence accumulation was the best model across all four difficulty levels and there was no evidence for leaky integration either (Fig. 6a,b). In contrast, with the ExaM we found a different picture: the collapsing bound model was frequent among the participants (Fig. 6d). This was the case for the intermediate difficulty levels. For the easiest and hardest difficulty levels there was strong evidence for the standard accumulation (Fig. 6c,d). These results are congruent with the PPL results, where we found for the ExaM that the collapsing bound model provides for each condition a closer fit to the behavioural data than the standard accumulation model (Fig. 7). Furthermore, for the ExaM there was no evidence of leaky integration (Fig. 6c,d).

**Figure 8:**
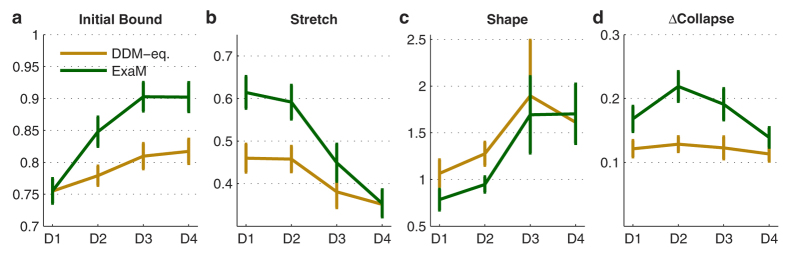
Collapsing bound parameters for DDM-equivalent model and ExaM. (**a**) Initial bound (**b**) stretch, which quantifies how much the bound will collapse (**c**) shape, which quantifies when the bound will collapse (**d**) the amount of collapse (calculated as the size of the initial bound minus the final bound value at *t* = *t*_*max*_).

**Figure 9:**
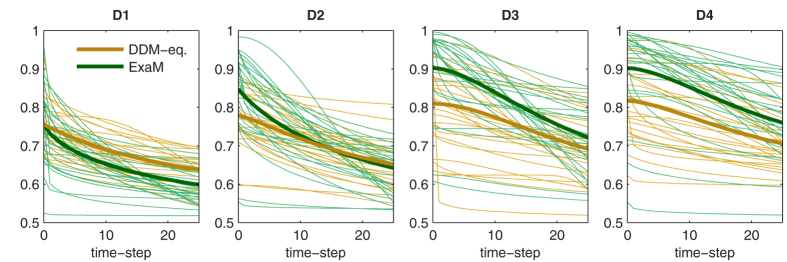
Collapsing bound shapes for both DDM-equivalent model and ExaM. Thin lines are individual participants (n = 24), thick lines are the bounds calculated from averaged bound parameters over participants.

In Fig. 8a–c, we show the three collapsing bound parameters (see Material & Methods), for both the DDM-equivalent model and the ExaM. These three parameters, the initial bound, the stretch (how far the bound falls), and shape (how early the bound falls) determine the overall effect of the collapsing bound. These parameters and their changes over conditions reveal how the collapsing bound may operate in our experiment: The initial bound (Fig. 8a) increases as the conditions get easier from D1 to D4 (Fig. 8b) for both DDM-equivalent model and ExaM. This effect is more prominent for the ExaM. For both the DDM-equivalent model and the ExaM, the stretch decreases as the conditions get easier. This means the overall effect of the collapsing bound (which also depends on the initial bound) is smaller for the easier trials. Again, this effect is more pronounced for the ExaM for all difficulty levels except for the easiest condition D1. For the shape parameter, we found that the bound drops earlier for the two most difficult conditions than for the two easiest conditions (See Material & Methods). See Fig. 9 for a visualisation of the fitted collapsing bounds.

Figure 8d shows the absolute amount of collapse for each difficulty level in terms of bound. Importantly, the amount of collapse closely resembles both the overall PPL pattern and the model frequency for the ExaM (see Fig. 6c,d and 7, number of trials under dotted vertical line). This indicates that both the PPL differences and model frequencies indeed capture the amount of collapse expected for different conditions.

**Figure 10:**
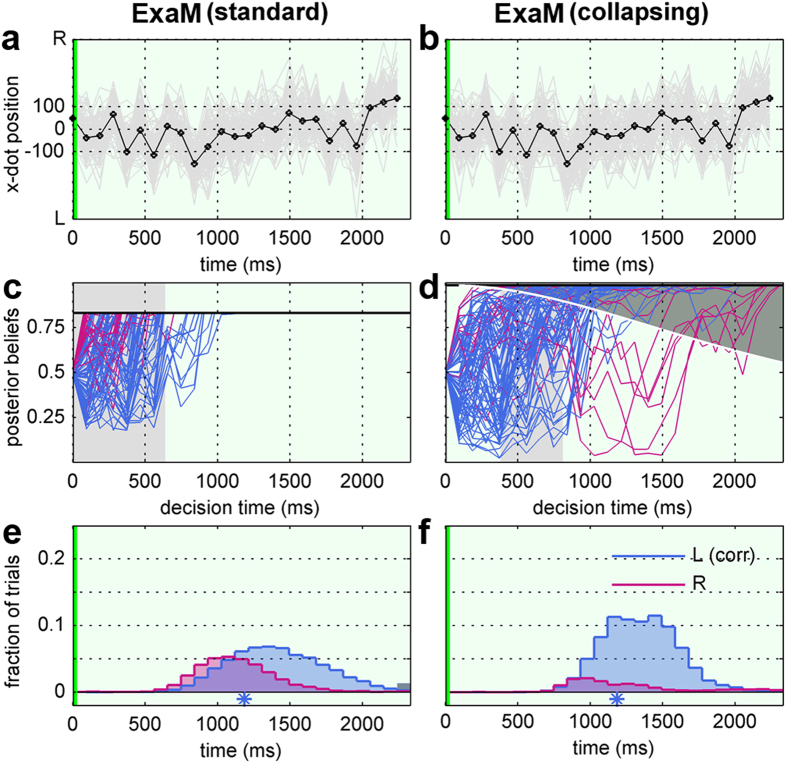
Single trial evidence accumulation and predicted RT distribution for standard evidence accumulation (standard) and collapsing bound (collapsing) for the exact input model (ExaM). A single trial from the second hardest difficulty level D2 from participant 2. Format as in Fig. 2. The shaded area in (**d**) indicates the collapsing bound.

Figure 10 shows an example of the evolution of the decision-making process under ExaM, with (Fig. 10b,d,f) and without (Fig. 10a,c,e) the collapsing bound from the same trial. Due to the first dot starting on the right side (Fig. 10a), early decisions tend to be the right alternative (Fig. 10c,e), with later decisions choosing the correct left decisions. However, with the collapsing bound, the initial bound is higher than the standard accumulation, thereby delaying the earlier decisions, which makes the erroneous right decision less frequent. This therefore increases the probability for the left decision, while reducing the probability for timed-out trials altogether (Fig. 10d,f). In other words, the collapsing bound shifts the probability mass from both early and late time points to more intermediate RTs.

## Discussion

In the present study, we have shown that modelling the detailed sensory input observed by the participants enables predicting response time and choice at the single trial level for a simple visual perceptual decision making task. We found that with the detailed sensory input model, a collapsing bound and its shape can be identified, providing a deeper understanding of the underlying decision making mechanism in humans. The single trial analysis may be especially useful when applied to neural measurements, because the model predicts trial-specific variations of internal, decision-related variables. This can be used for investigating the representations of these variables in the brain^33^.

When we compared the exact input model (ExaM) to the DDM-equivalent model, there were three main findings: Firstly, we found that the behavioural data, both choices and reaction times were better explained by the ExaM, both at the individual, single-trial level and on average. Secondly, the fitted noise parameter and non-decision time were significantly smaller for the ExaM compared to the DDM-equivalent model. This indicates that the DDM-equivalent model explained more of the behavioural data by unspecific noise processes, as opposed to the ExaM, which attributed more variability of the behavioural data to the actual decision making mechanism. Thirdly, the ExaM explained significantly more errors made by the participants than the DDM-equivalent model.

Our findings point towards a re-conceptualization of what constitutes an error: In the ExaM approach, there is no such label as correct/incorrect; rather we quantify how well such an apparently erroneous decision can be explained given the concrete sensory input seen by the participant (Fig. 2). This probabilistic way of modelling binary choices^34^ in combination with RT histograms may be a better basis for understanding the underlying mechanisms of decision making, than analysing average performance rates using only two classes of correct/incorrect decisions in conjunction with RT histograms reduced to five quantiles as typically done in standard analyses^12^,^13^.

Many experimenters in the perceptual decision making field use the ’random-dot motion (RDM)’ task rather than a single-dot tracking task as used here. The RDM task gained popularity after it had been used in perceptual decision making experiments with monkeys^10^,^35^. The original motivation was to employ a visual stimulus which is optimal for activating extrastriate areas, especially the motion-sensitive area MT, for neuronal recording^10^. In the present study, this was not the primary concern, but the aim was to design a stimulus that is most certainly perceived by the participant over all time points. This is important because with many dots as in the RDM task it is uncertain which dot movements the participants perceived and have used in their internal decision making. This uncertainty makes predicting behaviour at a single trial level challenging. By using single-dot stimuli, we minimized this uncertainty to showcase a proof of concept that precise modelling of the sensory input is useful for addressing specific questions about the underlying decision making mechanism. In our stimulus, the single dot location captures all variability whereas in standard RDM stimuli variability is distributed across many dots in the display. Thus, it is possible that we only observe a strongly predictable effect of the stimulus (tested with the ExaM), because participants attend more to the particular variability present in the single dot stimulus. This would also suggest that our paradigm is effective in isolating the mechanisms of decision making by reducing the uncertainty effects of lower level sensory processing, i.e., stimulus feature extraction. Note, however, that there is evidence that low-level features of RDM stimuli affect choices. For example, it has been found that the transient motion energy content of a RDM stimulus influences choices even in zero coherence trials, especially early in long trials^36^, or when only the moment-to-moment fluctuations not associated with the motion strength nor direction is considered^37^. Further, Bair and Koch^38^ have shown that the firing of MT neurons is temporally highly consistent across repeated presentations of exactly the same RDM stimulus. Because these MT neurons are thought to provide the evidence for decision making in RDM tasks^4^, this finding suggests that the particular, dynamically changing features of the RDM stimuli affect perceptual decisions. Indeed, when performing an analysis of the responses recorded in an RDM experiment (online database used in^11^, http://www.neuralsignal.org database nsa2004.1), we found that responses to RDM stimuli with “frozen noise”, i.e., with exactly the same sequence of dot movements, were markedly less random than responses to RDM stimuli in which the particular dot movements varied across trials (see Fig. S2). Stimulus details, therefore, also matter in RDM tasks, but it remains to be shown how much, especially, because it is still unclear exactly which spatiotemporal features the brain uses to judge the direction of RDM stimuli. In our study, we aimed to show that these results showing the correlation between noise and behaviour can be modeled and predicted by accounting for the exact sensory input. Although we did not collect data with ‘frozen noise’, we used the exact same stimuli for all 24 participants. In Fig. S3–6, the predicted RTs pooled over all participants are shown for all difficulty levels. These figures identify those trials (by the trial-specific PPL difference) for which most of the participants chose the incorrect targets. This further supports the notion that details in the input stimuli were inducing similar behavior across participants.

A recent auditory decision making study has modelled the precise timing of a sequence of click sounds to better resolve specific decision making parameters, as compared to standard analysis procedures^34^. The authors found that the main source of variability was based in the sensory input, and the accumulation was without leak. In addition to the same single-trial approach in this study, we also analysed reaction times: By only modelling participants’ choices, one may be limited in fully resolving the underlying parameters of decision making. In particular, for our design, the reaction time of a single trial determines how many dots have been seen by the participant and is therefore clearly relevant for modelling the observed choices. Furthermore, one implicit assumption of the ExaM is that participants weight single pieces of evidence (here, a single dot shown for 93 ms) by their relevance for deciding between the two alternatives^39^,^40^,^41^. For example, the more a dot’s location is to the left, the higher the likelihood for the left target. We were able to show that this weighting of evidence actually holds by finding that the ExaM is a better model than the DDM-equivalent model.

Although most theoretical accounts agree on the accumulation of evidence as a general mechanism of perceptual decision making^4^,^12^,^42^,^43^,^44^, it is currently unclear whether humans use a collapsing bound to conform to time pressures of decision making. In behavioural studies, positive evidence is sparse^22^,^45^ and has been established using standard DDMs with only few free parameters (two or three). Milosavljevic *et al.*^24^ showed that there is no advantage of the collapsing bound DDM once the standard (i.e., more complex) DDM^12^ is considered. In a recent study^25^, the authors conclude from a literature review and additional experiments that evidence accumulation with a fixed bound (but not a collapsing bound) is the most favoured model in perceptual decision-making, both in humans and in primates. One reason for this finding may be that the measurable effect of the collapsing bound on reaction times is rather small. The standard DDM approach is based on a severe reduction of data which aggregates the RT data into typically just ten RT quantiles per condition^12^ and comparing these with the model predictions as a measure of fitness. This data-reducing method may not be able to catch the subtle effect of the collapsing bound. Here, we have provided evidence that this question can be investigated with an increased modelling resolution by using a single-trial analysis.

We found that the ExaM with a collapsing bound better predicted participant behaviour than the other two model variants (Fig. 7). In addition, we found trends towards a collapsing bound model using Bayesian model selection for the medium difficulty levels (Fig. 6c,d). We only found evidence for a collapsing bound with the ExaM, when the exact sensory input entered the analysis.

The collapsing bound has been suggested as a mechanism to cope with the time limit to execute a decision^21^,^46^,^47^. Therefore, intuitively, harder trials should benefit more from the collapsing bound. However, our results indicate that in the most difficult condition the drop in bound is rather low (Fig. 8d). This may be due to an interaction between collapsing bound and the speed-accuracy trade-off chosen by participants across difficulty levels. Figure 8a shows that the initial values for the bound decrease as the task becomes more difficult. We also observed this effect in the models without collapsing bound (Table 1) where the bound is thought to implement the speed-accuracy trade-off chosen by the participant, see also^4^,^48^. This finding suggests that participants adapt their bound, i.e., the speed-accuracy trade-off, to the difficulty level of a trial which is indicated before the stimulus comes on (in our task, yellow dots are shown before white dot, Fig. 1a). Especially, participants may choose low bounds in difficult trials to counter small momentary evidences and an otherwise slow accumulation. Since the bound is already low at the start of a difficult trial, the effect of a collapsing bound in the hardest condition D1 is limited. In the easiest condition D4, the amount of collapse is the lowest (Fig. 8d). This result is fairly intuitive - the participants can make fast decisions without having to fear the upcoming end of the trial. If we used a paradigm which does not indicate the difficulty level at the beginning, we expect to see a higher initial bound at the most difficult D1 condition, and therefore more measureable collapsing bound effect, under the ExaM.

The shape of the collapsing bound (Fig. 9) determines when the bound drops strongest throughout the trial. We have found that difficult conditions exhibit an early drop, whereas in easier conditions there is a more gradual decrease. Although this does not seem to comply with the intuition that bounds should collapse towards the end of the trial, it is consistent with previous findings^21^,^22^. Clearly, more research is needed to investigate the causes of these particular shapes of collapse. Overall, we suggest that the ExaM is in principle sensitive enough to find evidence for a collapsing bound based on experiments, but the actual implementation of a collapsing bound by participants may depend on several factors, for example on the precise experimental design, the level of training, and instructions to the participants^25^,^49^.

In conclusion, modelling random spatiotemporal dynamics in the sensory input provides better fits of participant behaviour and enhances the predictive power of perceptual decision making models. Further, the increased modelling precision gained through incorporating the spatiotemporal dynamics of input stimuli into the model has allowed us to detect subtle effects of different decision making strategies in the behaviour.

In our present study we employed a particularly simple stimulus to maximally control the spatiotemporal information available for decision making. The proposed Bayesian account of evidence integration and computation can be easily adapted to different, more standard or more natural stimuli as long as there is high certainty about what features or parts of the stimulus participants is used to make decisions. For example, for the RDM stimulus motion energy^36^ could be used as spatiotemporal stimulus feature in our model. The described approach thus opens up a wide range of new experimental-computational studies with a large variety of stimuli to enable improved insights into the underlying mechanism of perceptual decision making.

## Material & Methods

### Participants

25 right-handed healthy volunteers participated in the study (mean age 24.8 years; 12 females). All had normal or corrected-to-normal vision, and reported no history of neurological or psychiatric diseases. Written informed consent was obtained from each participant. The experimental procedure was approved and carried out in accordance with the guidelines by the ethics committee of the University of Leipzig. One participant’s data were excluded due to the participant not being able to follow the instructions.

### Stimuli and Task

Visual stimuli were presented using the Presentation^®^ software (Version 16.5, www.neurobs.com) on a 1024 × 768 pixels CRT monitor (refresh rate: 75 Hz). Participants were seated in a chair ~70–80 cm from the monitor.

Stimuli were composed of one white moving dot, and two yellow stationary target position dots on a black screen (Fig. 1a). Two yellow target position dots were located mirror-symmetrically from the vertical midline of the screen. The distance of the two dots from the centre determined the difficulty of the trial (Fig. 1b). There were four difficulty levels where the two targets’ horizontal positions had a distance of 55 (easy), 40, 25 and 10 pixels (hard) symmetrically from the centre of the screen (corresponding visual angles: 3°, 2.2°, 1.4°, 0.6°, respectively). Each target position pair (e.g., −10, 10) was presented simultaneously on the screen.

The white moving dot was presented for 93.2 ms (7 frames) at a specific location (Fig. 1a), and jumped to a new position every 93.2 ms. Each position of the white dot was drawn from a Gaussian distribution with a mean equal to the target positions (left and right) and a standard deviation of 70 pixels. Each difficulty level had 200 trials (100 left + 100 right), resulting in a total of 800 trials per participant.

### Experimental Setup

The experiment consisted of three phases: 440 training trials with feedback followed by 40 rehearsal trials (without feedback) and finally the main phase with 800 trials (200 trials x 4 blocks, with breaks). In order to promote attentiveness and motivation, participants received a bonus for good performance (fast and accurate).

Before a trial started, a fixation cross was shown (duration: 300~500 ms). Next, two yellow target dot positions appeared for 700 ms, in addition to the fixation cross. Then the fixation cross disappeared (the yellow target position dots remained) and the moving white dot appeared (Fig. 1a). The task was to figure out around which target the white dot was moving. The stimulus was displayed until the participant reported the decision by pressing one of two buttons (left or right) on a 2-key button box, but maximally for 25 jumps of the white dot (≈2330 ms). If the participant failed to respond during this period, the trial timed-out and the next trial started.

### Analysis of Behavioural Data

All analyses were performed in MATLAB^®^ (version 7 or above, Mathworks, MA). The reaction time and response (left, right, timed-out) for each trial were recorded. Importantly, since the input stimuli were generated before the experiment, each participant saw the exact same 800 trials of white dot movements but in randomized trial order. Therefore, for each participant and trial, the precise visual input was available for subsequent analysis using the Bayesian model. The models were fit to the data five times and evidences showed little variation across EP-ABC repetitions. The models were fit to the actual response (left, right, timed-out) of the participants rather than the correct/incorrectness of the responses. Parameter estimates are shown in Table 1. The runs that gave the best fit to the data (largest model evidences) were used for further analyses.

We computed point estimates of choice and RT for each trial and each participant from the predicted response distributions. Specifically, in a given trial we chose the alternative (left, right) which was most frequent among 15000 simulations of that trial from the model with the fitted parameters. Further, we chose the corresponding RT as the most frequent time-point for the chosen alternative. Then, we did a simple match test between the real behavioural data and model predicted data (Fig. 5). For the RTs, we plotted the observed RTs against the predicted RTs as an indicator of a good match (Fig. S1).

### Experimental Stimulus

Input stimuli were generated by drawing dot locations for target *i*


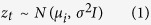


Where 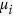 is the location of target 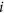, 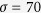 pixels is a standard deviation and 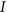 is the two-dimensional identity matrix. We set 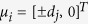
where 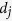 is a distance (in pixels) from the centre of the screen which is positive for target 2 (right), negative for target 1 (left) and the magnitude is one of 10, 25, 40 or 55 as determined by the condition (difficulty) of trial 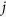.

### Bayesian Response Model

We recently presented a probabilistic Bayesian model which is equivalent to a pure drift diffusion model^16^. A major advantage of the Bayesian model is that it provides a direct link between stimulus and evidence accumulation whereas most other models of perceptual decisions abstract from the stimulus and only consider average effects of the stimulus on the evidence at any given point in time. We here exploit this property of the Bayesian model and simply deploy the mechanism we used to generate the stimulus for the experiment as generative model in the decision model. The Bayesian response model consists of: 1) input to the model (observations), (2) generative model(s) of the stimulus, (3) Bayesian inference as evidence accumulation and 4) decision criterion. We call 2–4 the decision model. Its free parameters are bias 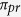 and confidence bound 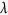, as described below.

Given the generative models and observed dot positions the decision model accumulates evidence for a target using Bayesian inference. Starting from a prior (bias) 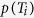, the decision model recursively computes the posterior beliefs 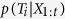 that the observed dot positions were generated from (one of) the two targets:


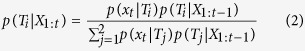


where 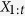 indicates all observed dot positions up to time point 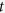 and for 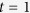 the previous posterior belief 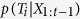 is replaced by the prior 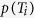. We parameterise the prior as the a priori probability that the dots were drawn from target 1, i.e., 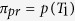. The a priori probability of target 2 then also derives from parameter 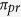 as 
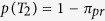
. 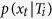 represents the generative model within the decision model. The generative models define the likelihood of dot positions under the hypothesis that these were generated from the corresponding target 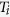. We assumed that the subjects had acquired a good representation of the possible stimuli after training and, consequently, used the same Gaussian distributions from which we created the stimuli as generative models. Specifically, we used 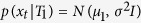 and 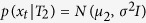.

The computed posterior beliefs 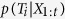
are the decision variables of the model. Consequently, the model makes a decision when either one of them reaches a set bound, i.e., when


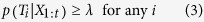


where 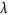 is a parameter of the model and can directly be interpreted as a level of confidence that a decision maker wants to reach before making a decision.

The target 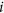 for which the posterior belief reached the bound is the choice of the model. The time 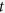 at which the bound was reached first is the decision time 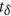 which is a discrete variable counting the number of observed dot locations. As described in the main text, the total reaction time of the model is the decision time plus a variable non-decision time 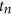 see, e.g.^12^,^50^, i.e.,


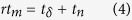


We modelled the non-decision time with a log-normal distribution to ensure positive non-decision times, i.e.,


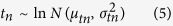


where 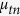 and 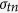 are again free parameters of the model.

We then transform 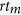 into a reaction time in millisecond, as recorded in the experiment, by multiplying with the time the single dot spent at the same location in the experiment, i.e., 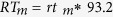 ms.

In the experiment, responses of subjects were timed out after a fixed deadline. We applied the same rule to the responses generated by the model, i.e., if 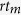 was greater than 25, the trial was timed out. The non-decision time (NDT) and non-decision time standard deviation (NDT SD) were calculated as the mode 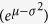 and standard deviation 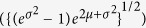 of the log-normal distribution with the fitted parameters 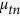, 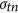 respectively.

Additional to these evidence-based decisions we allowed for random lapses in the model. We followed previous approaches to model lapses^22^ and randomly selected trials as lapse trials with probability 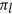 which was a free parameter of the model. When a trial was selected as lapse trial, it could either be a timed out trial, or a trial with random response. We set a lapse trial to timed out with probability 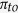 (free model parameter). With probability 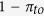 we then generated a random response by drawing choice and reaction time 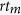 from a uniform distribution (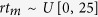 where 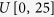 is the continuous uniform distribution within the range 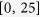.

In summary, the free parameters of the decision model were the prior probability of choosing target 1 (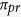), the bound on the posterior beliefs (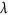), 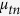 and 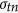 of the non-decision time log-normal distribution, the lapse probability (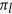) and the probability that a lapse trial is timed out (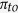).

### Input to the Decision Model

The Bayesian response model makes decisions based on observations which provide evidence for or against an alternative. We considered two kinds of observations reflecting different amounts of information about the true stimulus constituting the DDM-equivalent model and the exact input model (ExaM).

The observations containing most information about the stimulus are the dot locations themselves. Since we assumed that participants use the full information available on the screen (horizontal and vertical), we provided the 2D-coordinates of the moving dot in the ExaM as observations to the model: 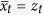. The DDM-equivalent model used minimal information about the time course of the presented stimulus, i.e., it used the fixed location of the true target as observations for the model: 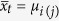 (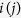 indicates the true target 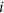 chosen in trial 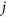). This is equivalent to how input is treated by previous perceptual decision making models, such as the drift diffusion model.

In both models we added noise to the mean (stimulus driven) observations 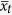 such that the actual observations which were input to the decision model were


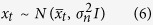


where 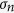 is the standard deviation of sensory noise which was fit to data together with the parameters of the decision model and 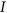 is the two-dimensional identity matrix.

### Inference Over Models Given Participant Responses

We used a second level of Bayesian inference to infer model parameters from behavioural data of participants. Standard inference methods require knowledge of the likelihood of the model for given data and model parameters. For the Bayesian response model, however, we chose a likelihood-free inference method. This approach has the advantage that we can easily modify the model without having to derive the potentially very complex likelihood function for the modified model. Yet, inference works as for the original model.

Likelihood-free inference methods are also known under the name of “Approximate Bayesian Computation” ABC, see^51^ for a recent review. Computation of likelihood values is skipped in these methods by simulating data points from the model and comparing simulated data points to the recorded real data on the basis of a summary statistic of the complete data set. As data points are simulated using a particular sample of parameter values (a sampled parameter set), ABC then rejects parameter sets incompatible with the data and approximates posterior distributions of model parameters based on the accepted samples. The main drawback of ABC methods is that inference is computationally very demanding, especially, when the statistic summarising the data set is high-dimensional.

Recently, a variant of ABC has been proposed which circumvents the use of summary statistics of the data^26^. The method extends an efficient approximate inference method (expectation propagation: EP) to the likelihood-free setting and is called EP-ABC. Instead of comparing whole data sets, EP-ABC compares sampled against measured data points individually while speeding up likelihood-free inference by orders of magnitude.

EP-ABC has been applied to a diffusion model^26^. We here use it for the proposed Bayesian model. A data point (response of participant or model) consists of the combination of choice (left, right) and reaction time of a single trial. Data points simulated from the model lead to acceptance of the corresponding parameter sample, if the simulated choice equals that of the participant for the given trial and the difference between the simulated reaction time and that of the participant is smaller than a parameter 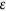. In all reported analyses we used 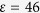 ms (half as long as the dot stays in a location on the screen in the experiment).

EP-ABC cycles through all data points in a data set where in our application each data point corresponds to the recorded behaviour of a single trial (choice and reaction time). After reaching a minimum number of accepted parameter samples for the given data point (set to 300 in our analyses), it updates the posterior distribution over model parameters and moves to the next data point. The updating scheme implements expectation propagation (EP)^52^,^53^ in which the true, potentially complex posterior distribution is approximated with a product of feasible distributions. In our application of EP-ABC each factor of this product represents one data point (trial) and is a Gaussian distribution. Updates of the posterior adjust the mean and covariance of one Gaussian factor such that a hybrid distribution and the full product of Gaussian factors become maximally similar. The corresponding hybrid distribution for one data point is obtained by replacing the Gaussian factor under consideration with the distribution estimated from the accepted parameter samples. Consequently, the main operation in the update of the posterior simply consists of estimating the mean and covariance of the accepted parameter values. In our analysis we used the implementation of this updating scheme provided by the first author of^26^ at https://sites.google.com/site/simonbarthelme/software/abcep_release.zip.

With increasing number of data points the posterior approximated by EP-ABC converges. To ensure convergence we made four passes through all data points of a data set. Additional to the posterior distribution over model parameters, EP-ABC also provides an estimate of the model evidence for the used model. The model evidence is returned as the log-marginal likelihood of the model given the whole data set.

We performed EP-ABC separately for trials from single conditions of individual participants, i.e., we obtained model evidences and parameter posteriors for each experimental condition of each participant. Therefore, EP-ABC data sets consisted of 200 trials. We had four different data sets per participant and we computed 24 × 4 parameter posteriors and model evidences per considered model.

**Figure 11:**
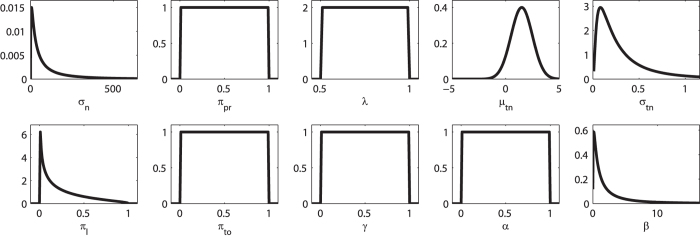
Prior densities for each parameter in Table 3. Note particularly the ranges of the distributions. Largest shown x-values of log-normal densities are the 0.95-quantile of the distribution.

Because we performed Bayesian inference over model parameters, we also needed to define parameter priors. EP-ABC approximates posterior distributions with Gaussians and, by default, also requires Gaussian priors. For simplicity we chose standard normal priors internally within EP-ABC. Because EP-ABC is based on sampling parameter values, we could, however, easily include parameter transformations which allowed us to implement various constraints for different parameter values leading to non-Gaussian priors for the parameters. In particular, we used two types of transformation: 1) exponential-transform and 2) uniform-transform. The exponential-transform maps a variable defined on the real line to positive values only: 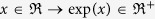. Thus, it transforms the Gaussian prior into a log-normal prior. The uniform-transform maps a normally-distributed variable through the cumulative density function of the standard normal distribution: 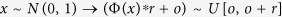. Thus, it transforms the Gaussian prior into a uniform prior on an interval defined by range 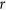
and offset 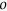. We list the resulting priors in Table 3 and the corresponding prior densities in Fig. 11.

**Figure 12:**
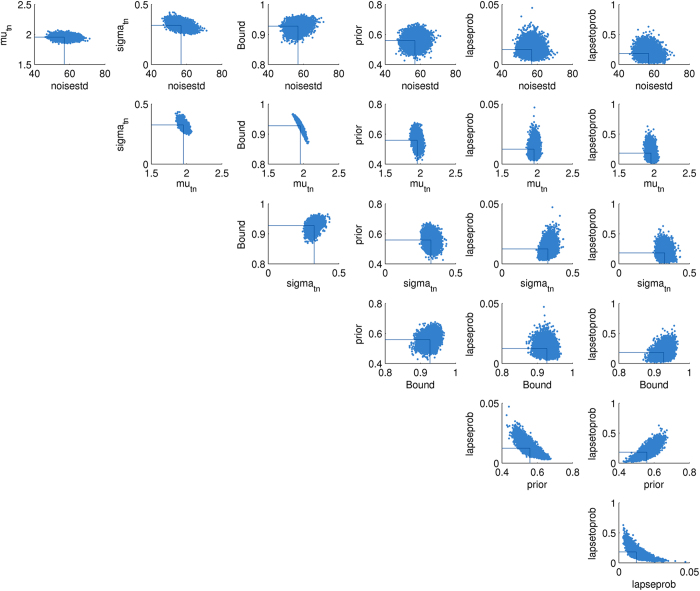
An example posterior parameter distribution. Each panel is a 2D-slice through the 7-dimensional parameter space (standard accumulation).

The posterior distribution over parameters inferred by EP-ABC for a given data set is a multivariate Gaussian distribution. Each dimension of that Gaussian corresponds to one parameter before its transformation. When reporting posterior parameter distributions, we report the distributions after transformation as estimated by sampling from the Gaussian EP-ABC posterior and transforming the samples according to the given transform functions. An example of a complete, sampled posterior distribution is shown in Fig. 12.

To compare models with different inputs and accumulation mechanisms, a random-effect Bayesian model selection (RFX-BMS) procedure as described by^27^,^28^,^29^ was used on model evidences produced by the ABC method. Protected exceedance probabilities were calculated with the VBM toolbox, available at: https://sites.google.com/site/jeandaunizeauswebsite/code/rfx-bms.

### Posterior Predictive Likelihoods

To quantify how good a model fits to participants’ responses, we computed the posterior predictive likelihoods. These quantify how likely a participant’s response is under the model after the model has been fit to the participant’s behaviour. Formally, the posterior predictive likelihood for a trial 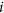
is the probability 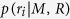 of the response 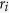 given the model 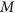 and the responses (choice and RT) of all trials 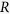.

We approximated the posterior predictive likelihood for a trial *i* by sampling 15000 parameter sets from the EP-ABC parameter posterior, transforming the parameter samples to the original parameter space and then sampling a response from the model for each of the 15000 parameter sets. From the 15000 sampled responses we estimated the response distribution of the model for trial 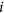. The posterior predictive likelihood is estimated as the fraction of sampled responses which are close to the participant response in trial 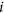
as determined by the EP-ABC distance criterion (matching choice and reaction time difference 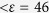 ms).

Since the posterior predictive likelihoods reuse the data to evaluate the model after adaptation to the data, they, in principle, may be too optimistic, when the model is too flexible and explains the noise in the data, i.e., when the model overfits. We have not found indication that the models we used overfit the data. Also, the models which only differ by the input (DDM-equivalent and exact input model) are equally complex. Comparisons based on the posterior predictive likelihoods, therefore, only reflect how beneficial knowledge of the corresponding input is for predicting the response of a participant.

### Evidence Accumulation Variants

We investigated two additional decision models which implemented two additional mechanisms previously considered in perceptual decision making: leaky accumulation^31^,^34^,^54^ and collapsing bounds^21^,^22^,^46^,^47^,^55^. Leaky accumulation departs from the optimal accumulation of evidence as defined by the Bayesian decision model by continuously degrading the influence of past evidence on the current beliefs.

We implemented leaky accumulation by using the following instead of Eq. (2):


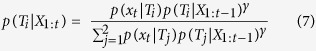


The exponent 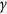 of the previous posterior beliefs is an additional free model parameter. Its effect is more clearly seen in the space of log-probabilities where we have


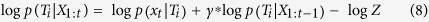



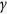
 controls how much influence the previous posterior beliefs have on the current beliefs. We also call it the discount factor. It varies between 0 (no accumulation) and 1 (perfect accumulation).

Motivated by electrophysiological findings e.g.,^21^ it has been suggested that decision variables are also influenced by ‘urgency signals’ which increase towards the end of a trial and push participants towards a decision independent of sensory evidence. Theoretically, an urgency signal is equivalent to a collapsing bound^22^. A collapsing bound captures participant’s willingness to trade accuracy against the chance to make a choice at all in light of an upcoming deadline. The overall shape of urgency signals, or collapsing bounds is still unclear. Although intuition dictates that bounds should collapse towards the end of a trial, empirical estimates tend to show an early drop in bound^21^,^22^. We here opted for a functional form for the bound which can represent both cases depending on its parameters:


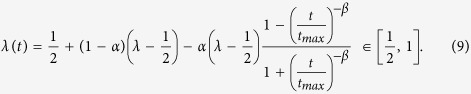


**Figure 13:**
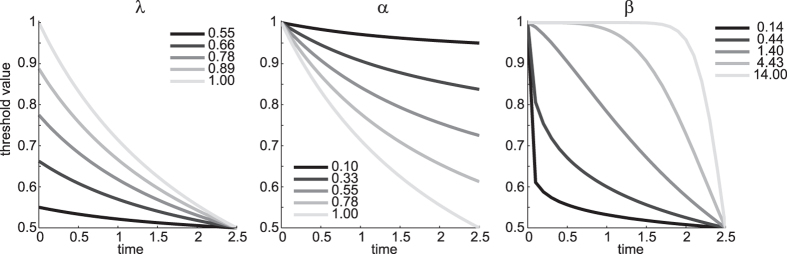
Demonstration of parameters of collapsing bound function. Each panel shows trajectories for varying values of one of the parameters 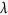, 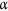 and 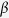. When one parameter was varied, the others were fixed at value 1.

This function, describing the time-varying bound, has three parameters. 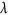 is the initial (maximal) bound value. 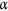 determines how far the bound collapses: for 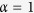 the bound collapses completely to the value of 0.5, for 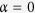
the bound does not collapse at all and stays at its initial value 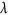. 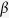 determines the shape of the collapse: for 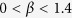 (approximately) the bound collapses early, for 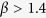 the bound collapses late. We demonstrate these effects in Fig. 13.

## Additional Information

**How to cite this article**: Park, H. *et al.* Spatiotemporal dynamics of random stimuli account for trial-to-trial variability in perceptual decision making. *Sci. Rep.* **6**, 18832; doi: 10.1038/srep18832 (2016).

## Supplementary Material

Supplementary Information

## Figures and Tables

**Table 1:** Mean posterior parameter fit for the DDM-equivalent model and exact input model over the four difficulty levels for 24 participants.

Parameter	DDM-Equivalent Model				Exact Input Model			
	D1 (hard)	D2	D3	D4 (easy)	D1 (hard)	D2	D3	D4 (easy)
Noise SD	236.70 (22.16)	76.90 (5.78)	70.73 (3.94)	51.29 (4.99)	95.63** (7.31)	58.27* (2.93)	52.10** (2.82)	38.22* (2.57)
NDT mode	563.26 (52.72)	453.31 (43.54)	431.24 (40.67)	430.51 (36.10)	520.73** (47.29)	415.96** (37.83)	382.16** (30.47)	399.35* (33.16)
NDT SD	195.17 (20.38)	121.61 (13.13)	137.60 (16.62)	164.97 (17.95)	201.66 (16.42)	181.27** (12.06)	172.54* (12.17)	167.54 (10.00)
Bound	0.71 (0.01)	0.74 (0.01)	0.83 (0.02)	0.83 (0.02)	0.65** (0.01)	0.77 (0.02)	0.87 (0.02)	0.88 (0.02)
Prior	0.46 (0.01)	0.46 (0.01)	0.46 (0.01)	0.43 (0.02)	0.47* (0.01)	0.47 (0.01)	0.44 (0.01)	0.42 (0.02)
Lapse prob.	0.03 (0.01)	0.03 (0.00)	0.02 (0.00)	0.03 (0.00)	0.02 (0.00)	0.02 (0.00)	0.02 (0.00)	0.02* (0.00)
Lapse (TO) prob.	0.24 (0.02)	0.16 (0.02)	0.18 (0.02)	0.14 (0.01)	0.24 (0.02)	0.17 (0.02)	0.18 (0.02)	0.15 (0.01)

TO = Timeout, NDT = Non-decision time, SD = Standard deviation. NDT mode and NDT SDs are reported in milliseconds. Bound and prior are both probabilities (range: 0 to 1). For the prior, 0 means complete right bias and 1 complete left bias. Values in parentheses are SE over participants. The asterisks indicate differences between DDM-equivalent model and exact input model for specific conditions (*p < 0.05, **p < 0.01, paired t-test over 24 subjects). Most notably, both the noise SD and NDT mode were higher for the DDM-equivalent model, for each condition. As expected, with increasing difficulty level the bound for making decisions decreases. The prior (i.e. a bias for reporting one of the two alternatives) indicates that participants slightly favour the right hand target. This is in line with the observed data; participants made on average approximately 5% more right decisions (data not shown).

**Table 2:** Model performances on predicting choice.

	DDM-Equivalent Model		Exact Input Model	
	participantcorrect	participantIncorrect	participantcorrect	participantincorrect
model correct	0.76	0.15	0.76	0.08**
model incorrect	0.05	0.04	0.05	0.11**

Proportion of correct and incorrect trials for DDM-equivalent model and exact input model (ExaM), pooled over all participants, and all conditions. The proportion of predicted incorrect trials was larger for the ExaM than for the DDM-equivalent model (**p < 0.01, paired t-test between DDM-Equivalent and ExaM over 24 subjects, and 4 conditions).

**Table 3:** Overview of free parameters in the Bayesian response model and its variants together with their prior distributions.

Model parameter	Prior	Prior parameters	Used by models
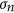 (noise std)	log-N	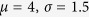	all
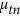 *	N	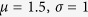	all
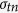 *	log-N	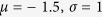	all
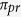 (prior)	uni	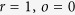	all
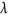 (bound)	uni	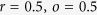	all
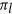 (lapse probability)	(cf. Fig. 11)	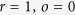	all
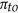 (time-out lapse probability)	uni	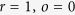	all
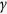 (discount rate)	uni	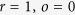	leaky
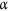 (stretch)	uni	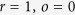	collapsing
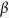 (shape)	log-N	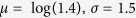	collapsing

log-N: log-normal prior with standard parameters 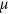 and 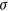. uni: uniform prior in interval 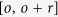. We chose these priors to cover all plausible parameter values while adhering to basic constraints such that standard deviation parameters must be positive and that parameters expressed as probabilities range between 0 and 1. For completeness, we show in Fig. 11 the resulting prior densities for each parameter. *: parameters of the log-normal distribution for the non-decision time.
