# Supplementary material for: Spatiotemporal dynamics of random stimuli account for trial-to-trial variability in perceptual decision making
Source: Sci Rep. 2016 Jan 11;6:18832. doi: 10.1038/srep18832 (PMC4707533; doi:10.1038/srep18832)
Supplement: Supplementary Information [file srep18832-s1.pdf]

Spatiotemporal dynamics of random stimuli account for trial-to-trial variability in perceptual decision making

Hame Park, Jan-Matthis Lueckmann, Katharina von Kriegstein, Sebastian Bitzer, Stefan J. Kiebel

Supplementary Information

Supplementary figures 1-6

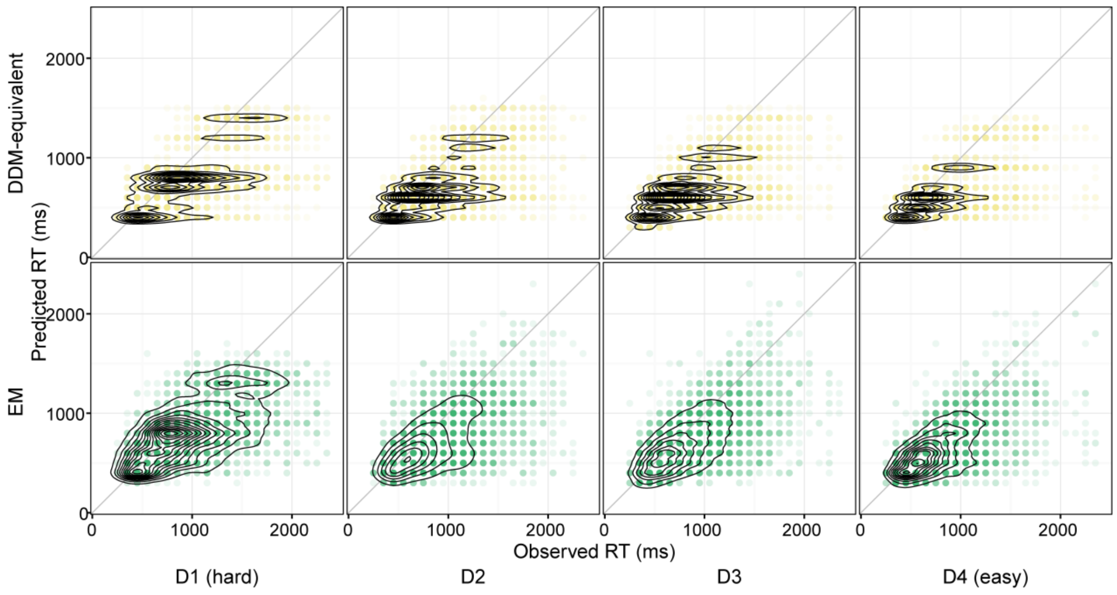

Figure S1. Comparison of DDM-equivalent model and exact input model (ExaM) in terms of RT point prediction accuracy. Experimentally observed RTs are plotted against predicted RTs. Only predictions that matched the observed choice were used for density calculation. Perfect predictions are on the diagonal grey line. Contour lines indicate regions of highest density. Across difficulty levels, the ExaM (green) predicts RTs better because the density outline of ExaM predictions more closely resembles an ellipse along the diagonal line. In the DDM-equivalent model RT densities (yellow), in turn, have less weight on the left side of the diagonal, i.e., they have a stronger bias towards providing predictions with short RTs.

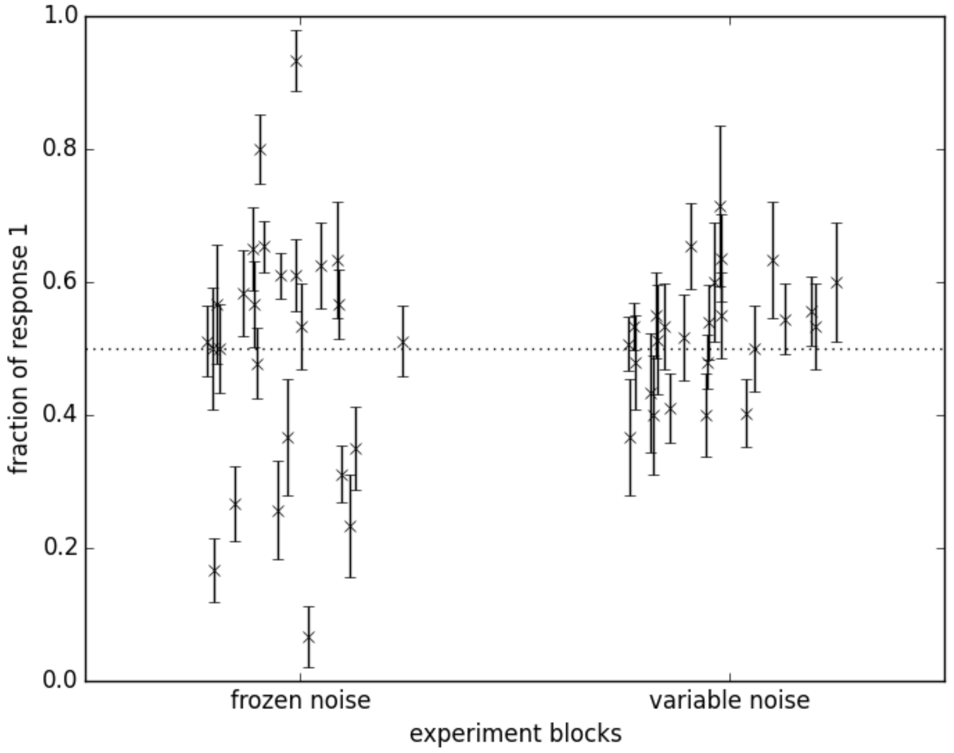

Figure S2. Effect of stimulus noise on decisions in a random dot motion experiment. We reanalysed data of [Britten KH, Shadlen MN, Newsome WT, Movshon JA (1992) The analysis of visual motion: a comparison of neuronal and psychophysical performance. J Neurosci 12:4745-4765] available at http://www.neuralsignal.org/data/09/nsa2009.1.html together with further information about the data. The data only contained 0% coherence trials of a single monkey. We show the fraction of trials in which one of the two possible responses was made. Each point shows the fraction together with its standard error for one block of trials. There were two different experimental conditions. In the 'frozen noise' condition (left) exactly the same random dot motion was shown on the screen in all trials of the corresponding block. In the 'variable noise' condition (right) the random dot motion varied across trials. We introduced random jitter along the abscissa within conditions to aid visibility. A fraction of 0.5 (dotted line) corresponds to the largest randomness in responses and fractions of 0 and 1 mean that always the same response was given in all considered trials. With frozen noise, responses were clearly more stereotyped than with variable noise, i.e., in 42% of the frozen noise blocks the shown fractions exceeded a distance of 0.15 from 0.5, while this was true for only 8% of the variable noise blocks. This indicates that in some blocks, the particular realisation of the random dot motion had a reproducible effect on responses. Note that other trials with non-zero coherence occurred between the 0% coherence trials in the experiment. Thus, it is unlikely that stereotyped responses resulted only from repeating the response of the previous trial. Our analysis is available online at https://github.com/sbitzer/RDMfrozenNoise.

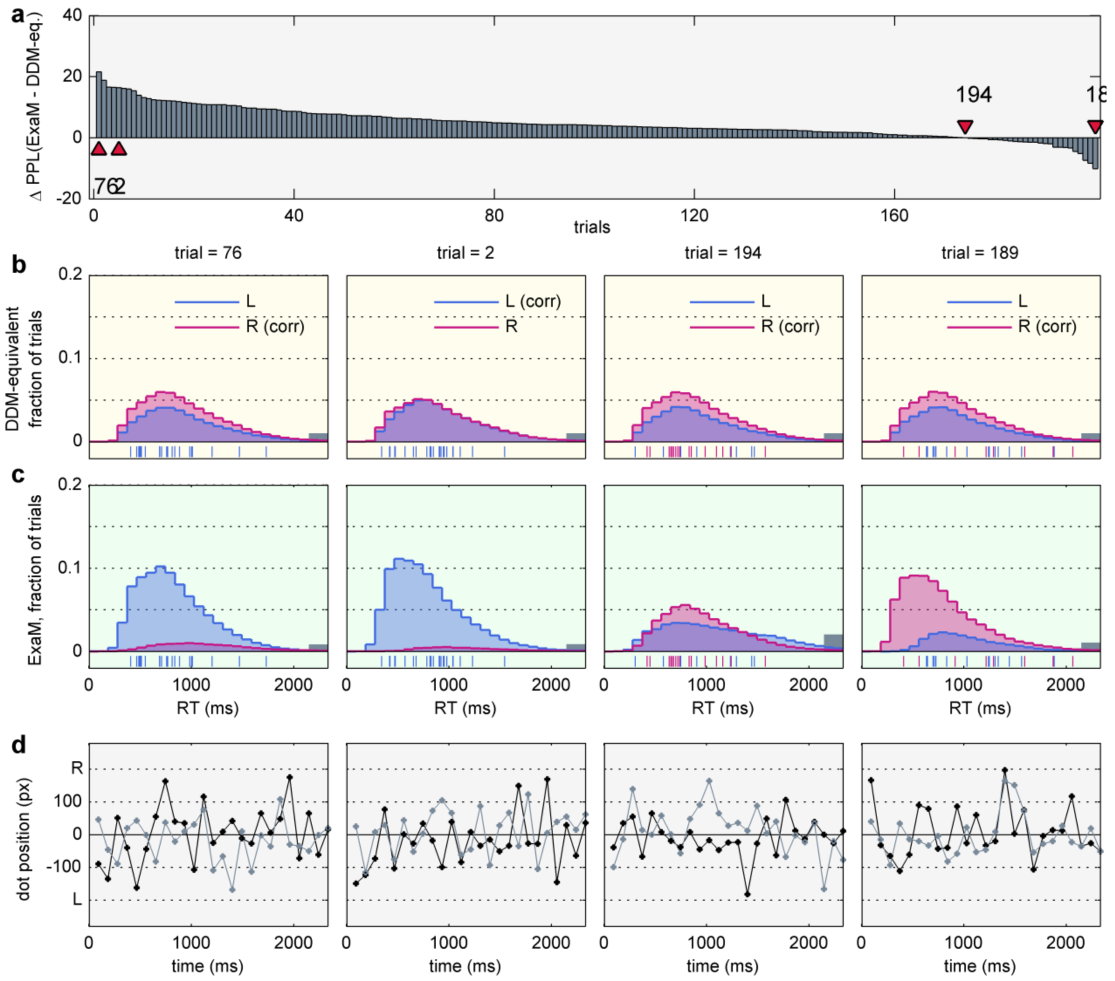

Figure S3. Illustrative example result: Predicted RT distributions for all participants, most difficult level (D1). **(a)** Predictive posterior log-likelihood (PPL) difference between DDM-equivalent (DDM-eq.) model and exact input model (ExaM) pooled over 24 participants. Red arrows indicate representative trials that were selected for further description. **(b)** Predicted RT distribution for selected trials from (a) under DDM-equivalent model. Blue and red bars indicate left and right responses respectively, vertical bars under histogram indicate real responses and RTs of all 24 participants. The correct target for each trial is indicated in the legend as 'corr'. **(c)** Same as (b) but under ExaM. **(d)** Horizontal (black: x-axis, gray: y-axis) dot positions of the input for each trial. **(b-d)** For the first trial, the ExaM gives a better explanation for most of the participants' behaviour since the actually observed dot trajectory starts with strong evidence for the left target, giving an early RT distribution for the left, followed by strong right evidence, which leads to RT predictions for the right. This reflects the observed responses of participants. The second trial presents very strong evidence for the left (correct) target so that most of the participants' responses were correct. This is captured well by the ExaM; the participant responses (vertical lines under plots) are clustered around the peak of the predicted RT distribution, and the DDM-equivalent model still predicts some right responses despite there being little evidence for this choice in the early stage of the trial. The third trial shows a case where both models have nearly the same PPL and give similar RT distributions. The fourth trial is an example of when the DDM-equivalent model gives a better prediction than the ExaM. It seems that the ExaM

treats the second data point as extremely strong evidence for the right target which prompts an immediate right response. However, this is not what participants seem to do. Most of them waited for more evidence emerging before committing to a decision.

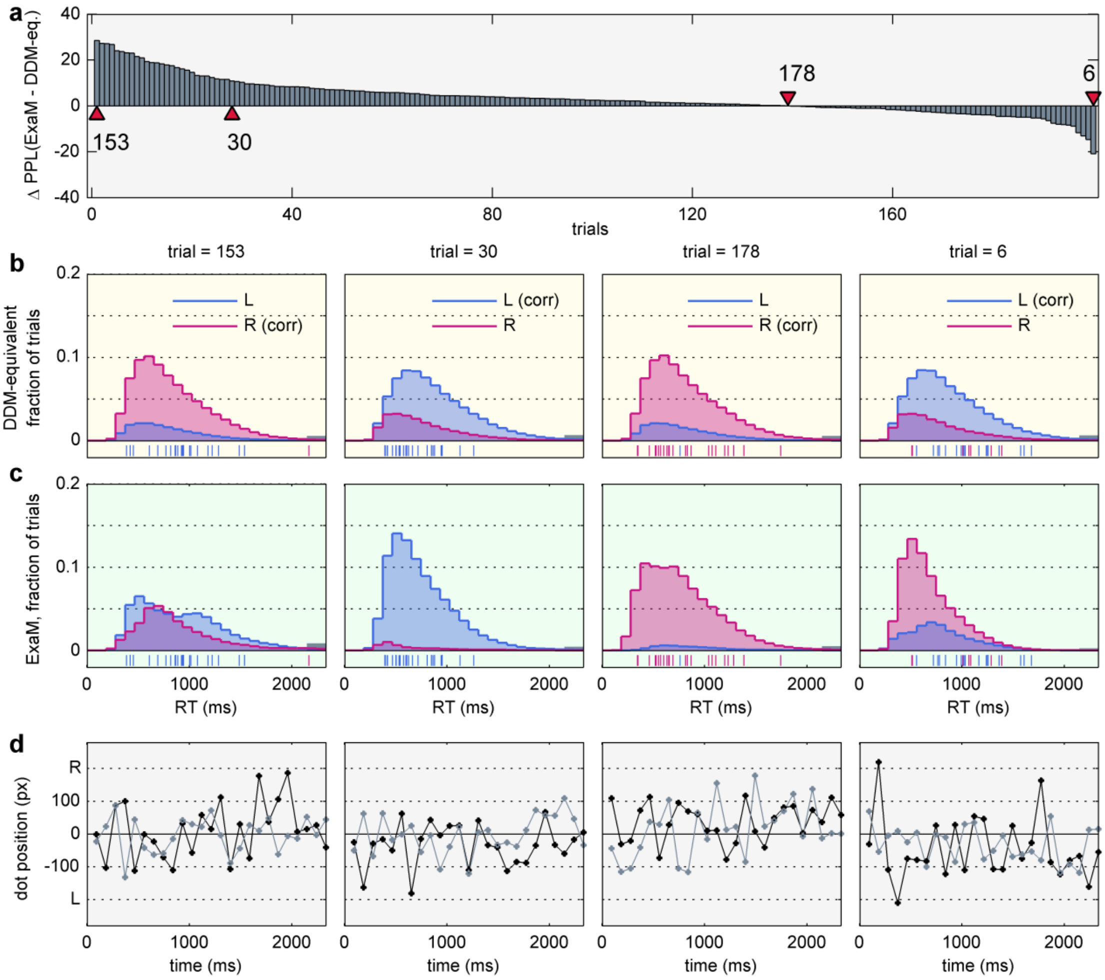

Figure S4. Illustrative example result: Predicted RT distributions for all participants, second most difficult level (D2). Same format as in Figure S3.

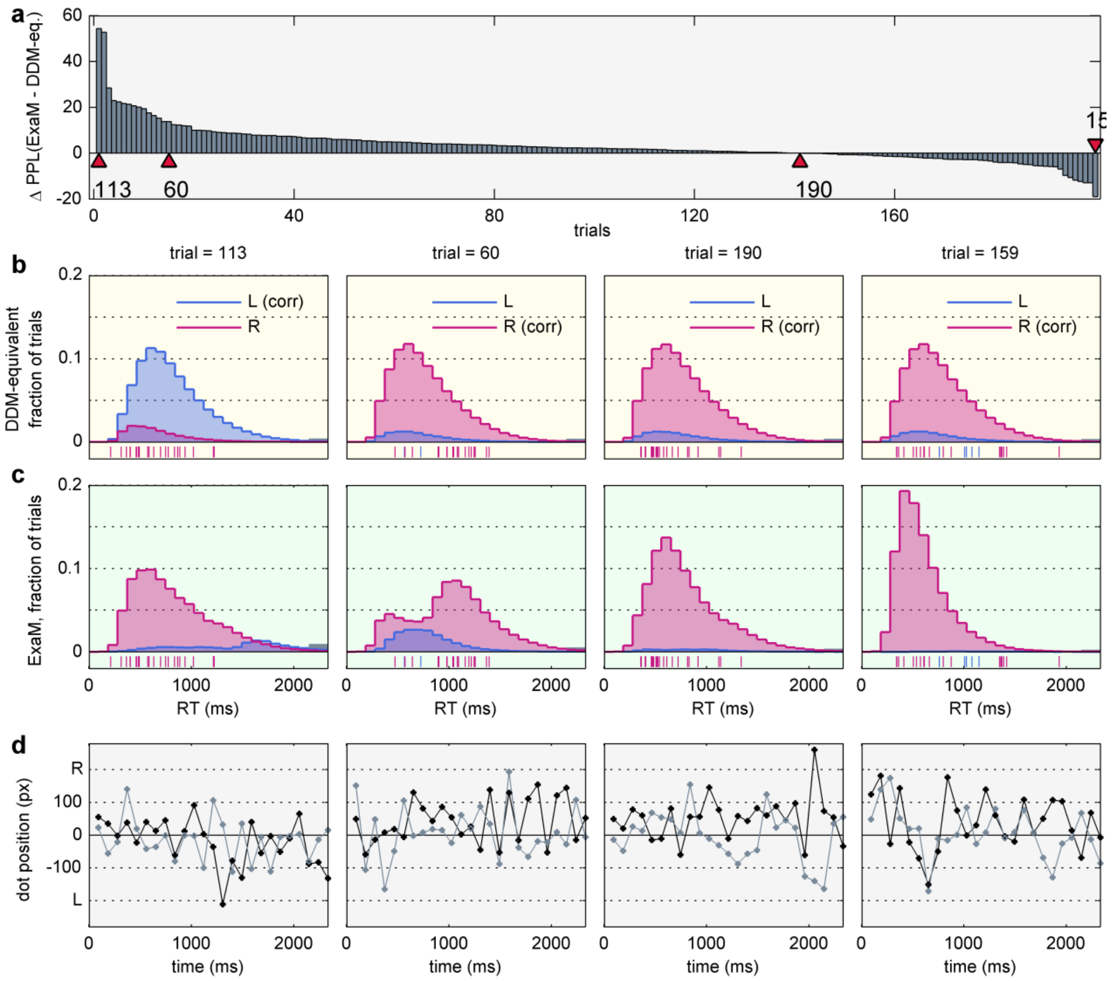

Figure S5. Illustrative example result: Predicted RT distributions for all participants, second easiest level (D3). Same format as in Figure S3.

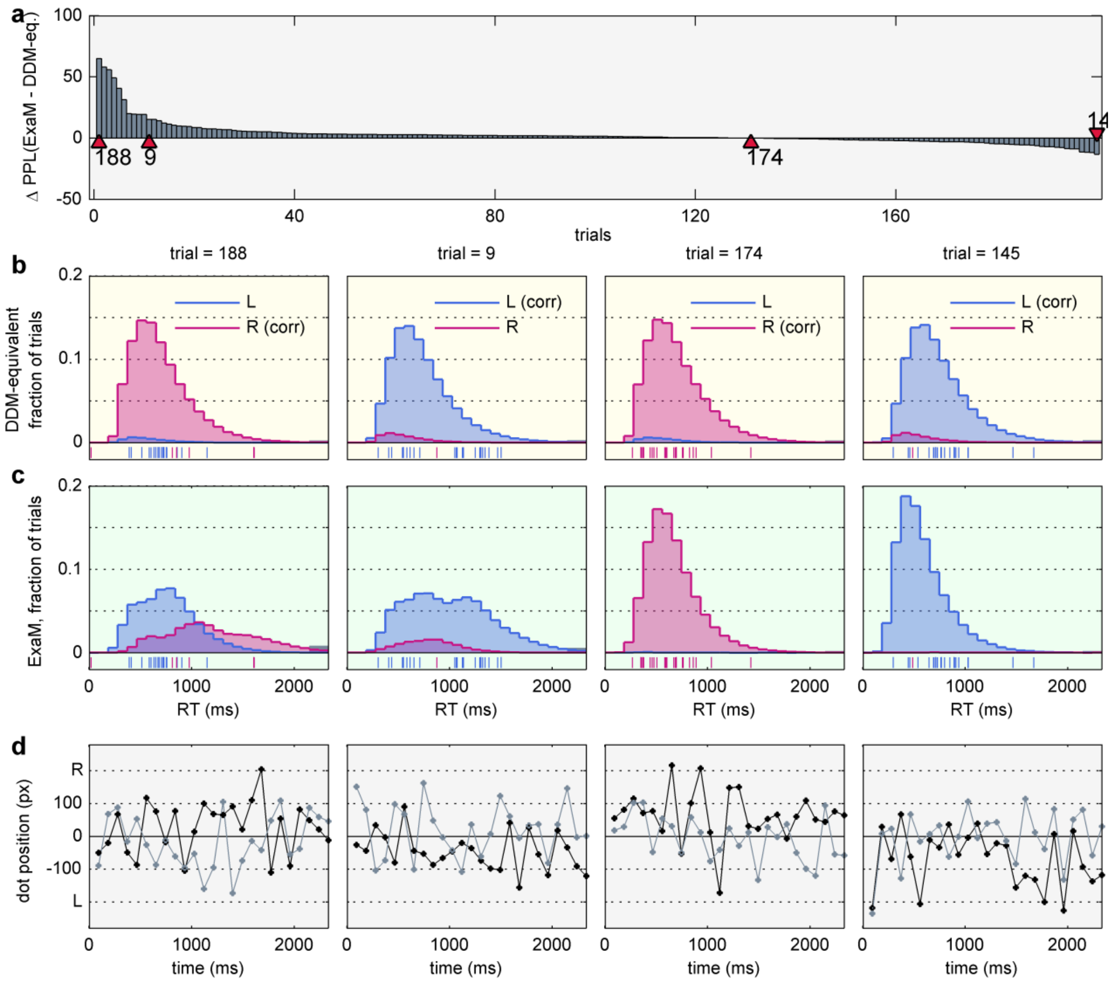

Figure S6. Illustrative example result: Predicted RT distributions for all participants, easiest level (D4). Same format as in Figure S3.
